# Trainee experiences of intellectual disability psychiatry and an innovative leaderless support group: a qualitative study

**DOI:** 10.1192/pb.bp.114.047373

**Published:** 2017-08

**Authors:** Ross Spackman, Hannah Toogood, Jayne Kerridge, Jon Nash, Elizabeth Anderson, Dheeraj Rai

**Affiliations:** 1 Dorset HealthCare University NHS Foundation Trust; 2 Avon and Wiltshire Mental Health Partnership NHS Trust; 3 Cambian Group; 4 King's College London; 5 University of Bristol

## Abstract

**Aims and method** There is very little research into the challenges of training in intellectual disability psychiatry or into interventions which may address these challenges. Using focus groups, we explored the experiences of intellectual disability psychiatry trainees, and evaluated a leaderless trainee support group developed in Bristol.

**Results** Five distinct themes were identified via framework analysis: that trainees felt unprepared for the difference from previous posts; the need for support; the value of the group; that trainees were concerned about judgement in supervision; that the group structure was valued.

**Clinical implications** Our findings highlight the support needs specific to intellectual disability psychiatry trainees. Leaderless peer support groups may be a valued resource to address such issues, and may be a useful model to be considered by other training schemes.

Intellectual disability psychiatry (also known as learning disability psychiatry particularly in UK healthcare services) is a specialty in the UK involving the assessment and management of mental health problems in individuals with intellectual disability and other developmental disabilities. Trainees in intellectual disability psychiatry face specific challenges, including the need to acquire enhanced communication skills, understand a range of genetic, neuropsychiatric and neurodevelopmental conditions, and utilise knowledge of complex psychopharmacology.^1^ They may also have to confront substantial existential and societal issues associated with caring for vulnerable individuals who face exclusion and disadvantage in many aspects of their lives.^2^

Although there is little published literature, one study exploring the experiences of intellectual disability psychiatry trainees described strong feelings of isolation, alienation, lack of skills and impotence, not unlike the feelings experienced by the patient groups the trainees worked with.^2^ Prompted by these findings, trainees on the Bristol intellectual disability psychiatry training scheme developed an innovative, leaderless trainee support group (Box 1) after taking advice from a consultant psychotherapist. The group aimed to provide a forum for reflective peer support for core and advanced intellectual disability psychiatry trainees (doctors specialising in psychiatry, previously termed senior house officers and specialist registrars). It has continued since 2005, with individual membership varying from 6 months (core trainees) to 3 years (advanced trainees).

Here we report the findings of a qualitative study exploring the experiences of trainees in intellectual disability psychiatry who participated in this support group. The aims were to explore doctors' experiences of training in intellectual disability psychiatry, in particular the positive and negative aspects of working in this specialty, and the role of support and supervision; and to evaluate the model of a leaderless peer support group from the perspective of its participants.

**Box 1** Trainee support group

- Who: All core and advanced trainees in intellectual disability psychiatry placements are invited. Typical attendance is 3–6 participants, dependent on need, leave or competing priorities. The group does not have a facilitator or leader, and was set up with an egalitarian structure.
- When: Fortnightly before the academic programme.
- Where: On the site of the academic meeting, central in the area between the different placements.
- How long: 90 min.
- Structure: 10 min of chat as people arrive, 70 min of focused support group. No fixed agenda, time used between trainees depending on needs that day.
- Boundaries: start and end time, confidentiality, respect.

## Method

We approached this project using a qualitative design, as we were interested in exploring a variety of individuals' interpretations of their experiences.^3^ Although one-on-one interviews were an alternative, focus groups provided a time-efficient way to gather data, allowed discussion of the issues raised, and enabled researchers to gain a sense of whether the individuals identified with each other's experiences.^4^

### Procedure

Two focus groups were held, one for each cohort, each lasting 90 min. Focus groups were facilitated by two of the researchers (J.N. and J.K. for the first cohort, and J.K. and D.R. for the second), who used a topic guide and encouraged free-floating discussion. The facilitators sought clarification when needed, and prompted the participants to move on to new areas when they started to repeat previously discussed issues, or after periods of prolonged silence. They also encouraged participants to share examples from their own work wherever possible. Recordings were transcribed, including spoken words, speech fillers and dysfluencies, by a medical secretary.^5^

The topic guide comprised four sections:

1. experiences of working with people with intellectual disability
2. the role of support and supervision in training in intellectual disability psychiatry
3. reflections on the value and usefulness of the trainee support group
4. specific consideration of the leaderless, egalitarian model of the trainee support group.

The list was generated from data gathered via a previous semi-quantitative survey investigating perceptions of the trainee support group held by trainees and trainers in 2007.

### Participants

All doctors who had been members of the trainee support group during the preceding year were invited to participate in each of the study's two focus groups. This process was performed twice, 4 years apart, sampling two non-overlapping cohorts. This was to allow exploration of whether experiences were cohort specific. Each focus group comprised five participants. The first included three core and two advanced trainees and the second two core and three advanced trainees. Each group was mixed gender and had trainees from different community learning disability teams (CLDTs) in the scheme. The ages of the participants were between 25 and 40 years.

Participation was voluntary, without incentive and occurred during work time. All participants consented to their comments being recorded and transcribed for analysis, with individual identities removed.

### Ethical considerations

The regional training programme director reviewed the proposal, considered relevant ethical issues and gave approval for the study.

### Analysis

Framework analysis was the most appropriate analytic method, given its relative simplicity and ease of use, and served the intended aims of seeking themes rather than generating explanations or new theories or concepts. A process similar to the framework analysis described in detail in Rabiee was followed.^5^

#### Stage 1

Two authors (J.K. and D.R. in the first and R.S. and H.T. in the second focus group) independently immersed themselves in the focus group transcript as a whole. The transcript was manually coded on paper, thus developing a ‘thematic framework’.

#### Stage 2

Following this, quotes were highlighted, pasted into a separate document and arranged by broad themes.

#### Stage 3

The interpretation stage of the analysis was done jointly between each pair of authors and involved refining and condensing the themed quotations using suggested criteria of: attention to frequency; emotion/intensity; specificity (attention to actual personal experience over hypotheticals); and extensiveness. Internal consistency (reduced attention to views subsequently contradicted or changed) could not be reviewed as suggested by Rabiee,^5^ as individuals could not be consistently tracked throughout the transcript.

#### Stage 4

The final stage involved a review of the analysed and interpreted data to see whether they had resulted in significantly overlapping themes suggestive of an overarching or superordinate theme. Themes were named by a process of abstraction as described by Fade.^6^ A requirement for reflexivity was acknowledged from the outset. Thus, researchers' influence was viewed as a necessity for making sense of the richness of the data generated by group participants rather than as bias to be eliminated.^7^

## Results

### Results of thematic analysis

Five distinct themes evident in both focus groups were identified. Owing to the anonymisation of participants during transcription, it was not possible to attribute quotes to specific participants. However, the quoted contributions incorporated a broad range of views arising in themes evident in both focus groups. Furthermore, it was evident in the transcript that they did not represent any one dominant voice.

#### Theme 1: unprepared for difference

Both groups perceived placements in intellectual disability psychiatry as being quite different to other psychiatry posts. This was viewed as having both positive and negative facets. Positive aspects included having more time allocated to conduct detailed assessments, and the potential to work jointly with colleagues from other professional groups. These were seen as enabling the doctor to assess the patient in a holistic manner and the overall experience was described as ‘rewarding’ by several trainees:

You don't just see the person in one dimension; you are seeing a holistic view.

Working with people with disabilities … can be very rewarding.

A variety of negative issues were discussed, including problems associated with reliance on suboptimal collateral histories and doctors questioning their own abilities to provide adequate care to this patient group, particularly with respect to specialist skills such as epilepsy management: ‘Suddenly you are asked to treat something you've probably not had a great deal of training for [epilepsy]… suddenly you are expected to already know about it.’ Some trainees appeared to be aware of such differences prior to starting posts, which was sometimes a source of apprehension. However, for others it was more of a surprise: ‘I think it is very different to what one gets in general psychiatry and I think that is not clear at the outset and sometimes can come as a surprise.’

A sense of isolation while working in these posts was part of the discourse in both groups. The change from being in large mental health teams to smaller CLDTs, where the other professionals have limited psychiatry training, was noted. The low number of medical colleagues in the CLDTs also generated feelings of isolation, as did the geographical spread of posts:

The posts can be quite isolating … there might be you and another doctor within the team.

We are quite isolated, we are far and few.

There is that huge geographical spread which means that you don't tend to see people informally.

#### Theme 2: need for support

The trainees described the need for help and support in coping with aspects of their work. For example, there were accounts of doctors struggling with the emotions evoked by working with a patient group with disabilities:

I think it is quite difficult to work with people with [intellectual disabilities]; especially people who are severely … physically and mentally disabled, it can make [you] feel quite low if you see those people who are completely dependent on carers.

If you work with someone who has so much pain and trouble, if you work with them and you don't get any support, I think I would personally get depressed, just thinking about it.

If you don't actually discuss your feelings with someone else who understands where you are coming from, I think that can pile on and on and can actually start affecting you in your personal life too.

Some trainees also described being overwhelmed by the additive effect of numerous emotionally challenging encounters: ‘In a week you collect things in your head. If there were deaths you would have a formal debrief, but these things are not deaths. They are things that keep on happening, small things.’

Many of the participants also expressed feelings of impotence to ‘fix’ the underlying conditions of their patients with intellectual disability: ‘I've always been taught to diagnose a disorder and treat it… I felt powerless.’

The trainees described the trainee support group and consultant supervision as addressing different support needs:

I think they are quite separate things… they do quite different things.

I think… supervision is about supporting your work and this [trainee support group] is a place that supports you.

The trainee support group was also highlighted by several trainees as a useful place to talk about difficulties related to training and difficulties experienced within CLDTs.

Being a doctor who is newly working in learning disability and the emotions that generates and the challenges of training, I think you get more out of discussing that with a group of people who are going through the same thing.

#### Theme 3: value of the group

The trainees described finding the group a source of:

Genuine support and encouragement and reassurance.

We talk, and when you talk it comes out, and you are able to share, and it is not so … painful anymore.

Some participants also recalled specific clinical and nonclinical issues where the group had been helpful to them: ‘I was struggling … the support was phenomenal.’ Trainees also stated that the group had a positive impact on their clinical work: ‘I think it does help us to become better clinicians in terms of how to deal with our emotions … we do learn from each other a lot.’

Positive aspects to the structure of the group included: permission to discuss anything, the group being confidential, and the opportunity to be with peers who are experiencing similar challenges:

The openness and the fact that you feel a bit equal… you can pretty much bring anything there.

I can speak and no one will judge me.

Sessions that had been of most value were reported to be those that were best attended, and setting ground rules was considered helpful to the group. If the group discussion was solely focused on an informal chat or issues such as rota swaps, it then lost its supportive benefits: ‘Incredibly valuable [sessions] have been the ones where people, lots of people, have come and come on time, and other times they definitely have felt like a missed opportunity.’

#### Theme 4: judgement in supervision

Participants described finding it easier to talk about their feelings with peers in the group than in consultant supervision. In particular, worries were expressed regarding looking incompetent during supervision, as the consultant would need to sign off the trainee at the end of the placement.

I don't want to say something [in consultant supervision] that will make me look bad, that will go on my file.

I would probably be worried in supervision that I don't want to say that I felt unsure about myself.

I find the trainee support group is more about me and about how I am coping, whereas clinical supervision is everything about the client and getting my assessments and appraisal.

Part of the issue might be your unhappiness with your interaction with other members of the team or with your consultant, which … you would find difficult to discuss in supervision.

However, consultant supervision sessions were deemed more appropriate for some other issues, which trainees said they would not discuss in a group setting: ‘Certain personal and professional issues that you may want to discuss in a supervision environment I wouldn't do in a Balint group or here.’

### Theme 5: group structure

The egalitarian model was described as enabling core and advanced trainees to express their opinions knowing trainees were viewed as of equal value to the group: ‘My views were valued and … I could also give advice to my senior colleagues, which is not always respected everywhere, so this was a major strength.’

It also allowed all members to talk about what they felt was important to them rather than to a facilitator. However, some noted a downside that a less confident member might not highlight their desire to bring a new topic to a session, and the group could be dominated by particular individuals:

There is a freedom in the group that comes from the fact that it's unstructured and doesn't particularly have an agenda.

I think a chairman would be useful [… ] in asking if particular quiet members would [… ] like to say anything because there are some people who have attended and I haven't heard speak in 6 months.

The lack of a leader was thought to promote a more lax view on attendance and punctuality, and some trainees and consultants were reported as giving the group a lower priority than other aspects of the trainees' work. Group members arriving late or leaving early was disruptive and disturbing: ‘We value the group, we see it as valuable or we wouldn't come at all, but we don't value it as highly as other things in our timetable so it tends to be the first thing that gets bumped.’

## Discussion

This study adds substantially to the very limited literature detailing the peculiarities and challenges of training in intellectual disability psychiatry.^1,2^ One strong theme that emerged from our results was how trainees considered training in intellectual disability to be different from other psychiatric posts and the degree to which they were prepared for this. The reasons cited were related to both the specifics of the work and the structure of teams. It is well known that psychiatric disorders in intellectual disability may be more complex to diagnose, particularly owing to difficulties in effective communication. A further contrast with many other areas of psychiatry is the degree to which healing or restoration to full function or participation is possible. In intellectual disability psychiatry, the primary disability is often the intellectual impairment or associated developmental disorder, thus treatment of any mental illness may restore the patient's previous level of functioning and quality of life, but no further. Despite epilepsy management being a common role for the intellectual disability psychiatrist,^8^ our findings suggest that many trainees felt apprehensive about their skills and confidence in this area.

Feelings of isolation were also highlighted. CLDTs in the area of the study are geographically spread out and based away from their mainstream psychiatric colleagues. Separation from peers has been noted as off-putting to foundation doctors,^9^ but few studies explore feelings of isolation among psychiatry trainees^2^ It has been noted previously that individuals who work with people with disabilities can feel stigmatised and isolated.^10–12^ Stigma by association is the process by which relatives, support staff, friends and associates feel stigmatised owing to their contact with a stigmatised group.^13,14^ This may also affect trainees working in this area and contribute to their feelings of isolation. We think that one reason the group was valued could be its ability both to reduce the feeling of isolation by bringing trainees together, and to mitigate some of the stigma felt by enabling the trainees to share difficult experiences. One could speculate that the group may have not just attenuated some potential negatives of the subspecialty training, but also contributed to the enjoyment and reward of it. If this were to be true, it would be interesting to study whether training schemes in areas with specialty-specific support or educational groups do better in relation to trainee retention or satisfaction than those without such structures.

It should be noted that despite the challenges, there was also a strong and pervasive feeling of positivity about training and working in intellectual disability psychiatry. Such experiences were startlingly absent in previous work,^2^ but are important to note to reassure future recruits in the specialty. In particular, the trainees mentioned the term ‘rewarding’, a varied and complex concept.^15^ The view that community-based intellectual disability psychiatry would be rewarding was predicted some 30 years ago,^16^ although this is the first study as far as we are aware that affirms this view. Intellectual disability requires a particularly holistic approach, often not dissimilar to the approach of general practitioners (GPs). While there is an absence of published surveys or qualitative studies on what psychiatrists find rewarding, interpersonal relationships between doctor and patient have been found to be particularly satisfying for GPs.^17^ However, we are unaware of similar studies among psychiatrists.^18^

Both focus groups discussed how consultant supervision and the support group were different, but mutually supportive and compatible. When surveyed, UK trainees report they are mostly happy with supervision and find it useful.^19^ The Royal College of Psychiatrists recommends that supervision should enable ‘the development and assessment of clinical and personal skills under direct one-to-one supervision by an expert’ and should be ‘focused on discussion of individual training matters’.^20^ The hierarchical nature of consultant supervision is both valued by trainees^21^ and necessary for valid competency assessments, but this can be a potential barrier to seeking support in some areas, particularly revealing vulnerabilities to a supervisor who is also an assessor. Personal upset and secondary grief relating to patients is considered by some supervisors and supervisees to be a boundary breach in supervision.^22^ The trainee support group is set up without hierarchy and this may have contributed to reducing boundaries around discussing vulnerabilities. Honest discussion about how trainees were ‘coping’ was easier in the trainee support group than supervision, despite the College suggesting consultant supervision should include this.^20^ This study adds to previous work suggesting some mismatch between intent and what trainees feel comfortable discussing^23^ This space for honesty is a value of the group but it would be a concern if the availability of the trainee support group and its support acted as a colluder or barrier to honesty in consultant supervision.

Group peer support may be more beneficial than alternatives such as paired peer support. Several of the benefits described in both focus groups suggested similarities to Yalom's therapeutic factors: universality, altruism, guidance, imparting of information, cohesion, and existential factors.^24^ That senior trainees stayed in the group for up to 3 years may reinforce some of these factors, and their relative maturity and existing trust following a longer involvement in the group may provide additional support.^25^ It also provides senior opinions, which have been found to be supportive when shared in other contexts.^26^ A common concern when leaderless groups are used for supervision is a loss of focus on the task and drifting into support and advice-giving.^27,28^ As the model presented here is primarily for support, and advice is part of that, the lack of leader is not a concern in this context, although the results did suggest that some participants would have liked a facilitator role in encouraging quieter members to contribute.

As this is an analysis of a single group and the findings have not been replicated elsewhere, it is difficult to generalise the utility of such groups to other areas. However, we think similar models of egalitarian peer support that require limited resources for setting up may have the potential to benefit trainees in other, smaller or more challenging, specialties.

### Strengths and limitations

The qualitative design and use of focus groups is appropriate to investigate attitudes and experiences of trainees. The training rotation is relatively small, with 7–9 core and advanced trainees available to attend the trainee support group in each 6-month period. ‘Group think and the articulation of group norms may have introduced a positive bias. However, the anonymity of participants in the transcript is likely to have mitigated self-censorship and there was evidence of a diversity of opinion, particularly illustrated by both praise and criticism of the trainee support group and highlighting a range of experiences in training. The anonymisation happened at transcription rather than at analysis stage. This precluded the ability of the authors analysing the data from tracking individuals’ comments or reviewing whether they were linked to particular posts or trainers. Generalisability is a concern in qualitative studies, and was also a concern in this particular training scheme. First, at the time of the focus groups, the Bristol scheme was performing above average on trainee satisfaction in GMC surveys.^29^ Second, many of the CLDTs may be unusually isolating for trainees compared with elsewhere in the country by virtue of their geographic spread and relatively rural setting. Finally, in several CLDTs within the scheme, most team members, except psychiatrists, are employed by a different organisation and may have few psychiatric skills.

In conclusion, we have highlighted some of the challenges and rewards of training in intellectual disability psychiatry. Our evaluation of an egalitarian, trainee-led peer support group suggests that the model could be useful for other intellectual disability psychiatry training schemes. Whether this could be a support structure suitable for other specialties remains to be studied.
